# Conservation and divergence of transcriptomic and epigenomic variation in maize hybrids

**DOI:** 10.1186/gb-2013-14-6-r57

**Published:** 2013-06-12

**Authors:** Guangming He, Beibei Chen, Xuncheng Wang, Xueyong Li, Jigang Li, Hang He, Mei Yang, Lu Lu, Yijun Qi, Xiping Wang, Xing Wang Deng

**Affiliations:** 1 Peking-Yale Joint Center for Plant Molecular Genetics and Agro-Biotechnology, State Key Laboratory of Protein and Plant Gene Research, School of Life Sciences, Peking University, No. 5 Yiheyuan Road, Haidian District, Beijing 100871, China; 2 Department of Molecular, Cellular and Developmental Biology, Yale University, 165 Prospect Street, New Haven, CT 06520, USA; 3 Institute of Biophysics, Chinese Academy of Sciences, 15 Datun Road, Chaoyang District, Beijing 100101, China; 4 Tsinghua-Peking Joint Center for Life Sciences, School of Life Sciences, Tsinghua University, Tsinghua Park No. 1, Haidian District, Beijing 100084, China; 5 National Key Facility for Crop Gene Resources and Genetic Improvement, Institute of Crop Science, Chinese Academy of Agriculture Sciences, No. 12 Zhongguancun South Street, Haidian District, Beijing 100081, China; 6 College of Life Sciences, Beijing Normal University, No. 19 Xinjiekouwai Street, Haidian District, Beijing 100875, China; 7 National Institute of Biological Sciences, No. 7 Science Park Road, Zhongguancun Life Science Park, Beijing 102206, China

**Keywords:** maize hybrids, transcriptome, epigenome

## Abstract

**Background:**

Recent genome-wide studies suggested that in addition to genetic variations, epigenetic variations may also be associated with differential gene expression and growth vigor in plant hybrids. Maize is an ideal model system for the study of epigenetic variations in hybrids given the significant heterotic performance, the well-known complexity of the genome, and the rich history in epigenetic studies. However, integrated comparative transcriptomic and epigenomic analyses in different organs of maize hybrids remain largely unexplored.

**Results:**

Here, we generated integrated maps of transcriptomes and epigenomes of shoots and roots of two maize inbred lines and their reciprocal hybrids, and globally surveyed the epigenetic variations and their relationships with transcriptional divergence between different organs and genotypes. We observed that whereas histone modifications vary both between organs and between genotypes, DNA methylation patterns are more distinguishable between genotypes than between organs. Histone modifications were associated with transcriptomic divergence between organs and between hybrids and parents. Further, we show that genes up-regulated in both shoots and roots of hybrids were significantly enriched in the nucleosome assembly pathway. Interestingly, 22- and 24-nt siRNAs were shown to be derived from distinct transposable elements, and for different transposable elements in both shoots and roots, the differences in siRNA activity between hybrids and patents were primarily driven by different siRNA species.

**Conclusions:**

These results suggest that despite variations in specific genes or genomic loci, similar mechanisms may account for the genome-wide epigenetic regulation of gene activity and transposon stability in different organs of maize hybrids.

## Background

The chromatin states and genome activity in eukaryotes are regulated by a variety of different epigenetic mechanisms, mainly DNA methylation, histone modifications, and the RNA interference pathway [1-3]. DNA methylation, that is, the addition of a methyl group to a cytosine by DNA methyltransferases, is primarily regarded as a relatively stable repressive epigenetic marker, which maintains genome stability by suppressing the activity of transposons and other repetitive sequences [4,5]. Recent studies have also indicated an additional potential role for DNA methylation in regulating the expression of protein-coding genes [6-8]. In some cases, the methylated cytosines can be removed by DNA glycosylase [5]. Histone modifications, which are post-translational modifications of histone proteins at their N-terminal tails, provide a dynamic and reversible mechanism to regulate gene expression in response to diverse endogenous and exogenous stimuli [9,10]. Lysine acetylation (for example, H3K9ac) and some lysine methylations (for example, H3K4me3 and H3K36me3) are associated with transcriptional activation of genes [8-11]. Small RNAs, especially small interfering RNAs (siRNAs), provide another layer of epigenetic regulatory mechanism, and repeat-associated siRNAs are known to be involved in the maintenance of genome stability by RNA-directed DNA methylation (RdDM) [1,12,13].

Recently, genome-wide studies of these epigenetic components using high-throughput approaches have identified complex networks of their variations during plant evolution and development. For example, some studies characterized the patterns of DNA methylation, histone modifications, and small RNAs (sRNAs) in various tissues or organs of plants [7,14,15], whereas other studies compared the DNA-methylation patterns between different plant species or different genotypes [16-20]. Briefly, these studies revealed the conservation and divergence of epigenetic components in different plant organs or genotypes. Moreover, some studies also discovered the genome-wide epigenetic variations and their potential relationship with altered chromatin states and changed gene activity in plant hybrids. Fox example, the global DNA-methylation variations and their potential association with altered gene expression in hybrids have been extensively discussed [8,21-25]. Further, sRNAs were also found to show extensive variation in hybrids of *Arabidopsis *[21,24-26], rice [8,23,27] maize [28], wheat [29], and yellow poplar [30], and altered siRNA levels were presumably associated with changed DNA methylation in hybrids through the RdDM pathway [21,23-25].

Maize is an appropriate model organism for studying global genetic and epigenetic variation in plants because of its exceptionally complex genome (in particular the high content of transposable elements (TEs)) and is a particularly rich source of epigenetic discoveries [31]. Recent studies have explored the global transcriptional variations in maize hybrids in various tissues or organs, including embryos [32,33], endosperms [33], immature ears [34], leaves [35], stem meristem [36], seedling shoots [37,38], and roots [39,40]. However, the mechanisms underlying these variations and their conservation and divergence between organs remain elusive. In addition, global epigenetic profiling in maize showed that siRNA populations vary following hybridization, and are associated with regulated transposons in the seedling shoot apex and developing ear of maize hybrids [28], and that DNA methylation is associated with allelic expression of imprinted genes in the endosperm of maize hybrids [22]. Therefore, it is necessary to comprehensively determine both the genome-wide epigenetic variations and their association with transcriptional divergence between different organs of maize hybrids.

In this study, we generated highly integrated maps of transcriptomes and epigenomes in shoots and roots of two maize inbred lines and their reciprocal hybrids, and obtained a comprehensive view of the variation in gene expression, DNA methylation, histone modifications and sRNAs between organs and genotypes. In general, histone modifications are associated with differential gene expression between organs and between hybrids and parents, whereas, siRNAs and DNA methylation are mainly associated with regulated TEs and other repetitive elements, and thus may change the chromatin states in hybrids. Despite the variation in specific genes or genomic loci, similar global trends of transcriptomes and epigenomes were seen in both shoots and roots of reciprocal hybrids. Our data therefore may serve as a useful resource to better understand the epigenetic basis of gene action in different organs and different genetic backgrounds.

## Results

### Transcriptomic and epigenomic profiling in shoots and roots of two maize inbred lines and their reciprocal hybrids

We used Illumina high-throughput sequencing approaches to generate integrated maps of mRNA and sRNA transcriptomes, DNA methylomes and genome-wide distribution of three representative histone modifications (H3K4me3, H3K9ac, and H3K36me3) in two maize inbred lines (B73 and Mo17) and their reciprocal hybrids (B73 ´ Mo17 and Mo17 ´ B73). Shoots and roots of both hybrids and parental lines from 14-day-old seedlings were used for all experiments in this study. Illumina sequencing libraries for mRNA sequencing (mRNA-seq), chromatin immunoprecipitation sequencing (ChIP-seq), *Mcr*BC sequencing (*Mcr*BC-seq), and sRNA sequencing (sRNA-seq) were constructed as previously described [3,7,8]. All sequencing reads were aligned to the reference genome of the maize inbred line B73 (ZmB73_RefGen_v2) [31] using Bowtie software [41] (see Additional file 1, Table S1).

**Figure 1:**
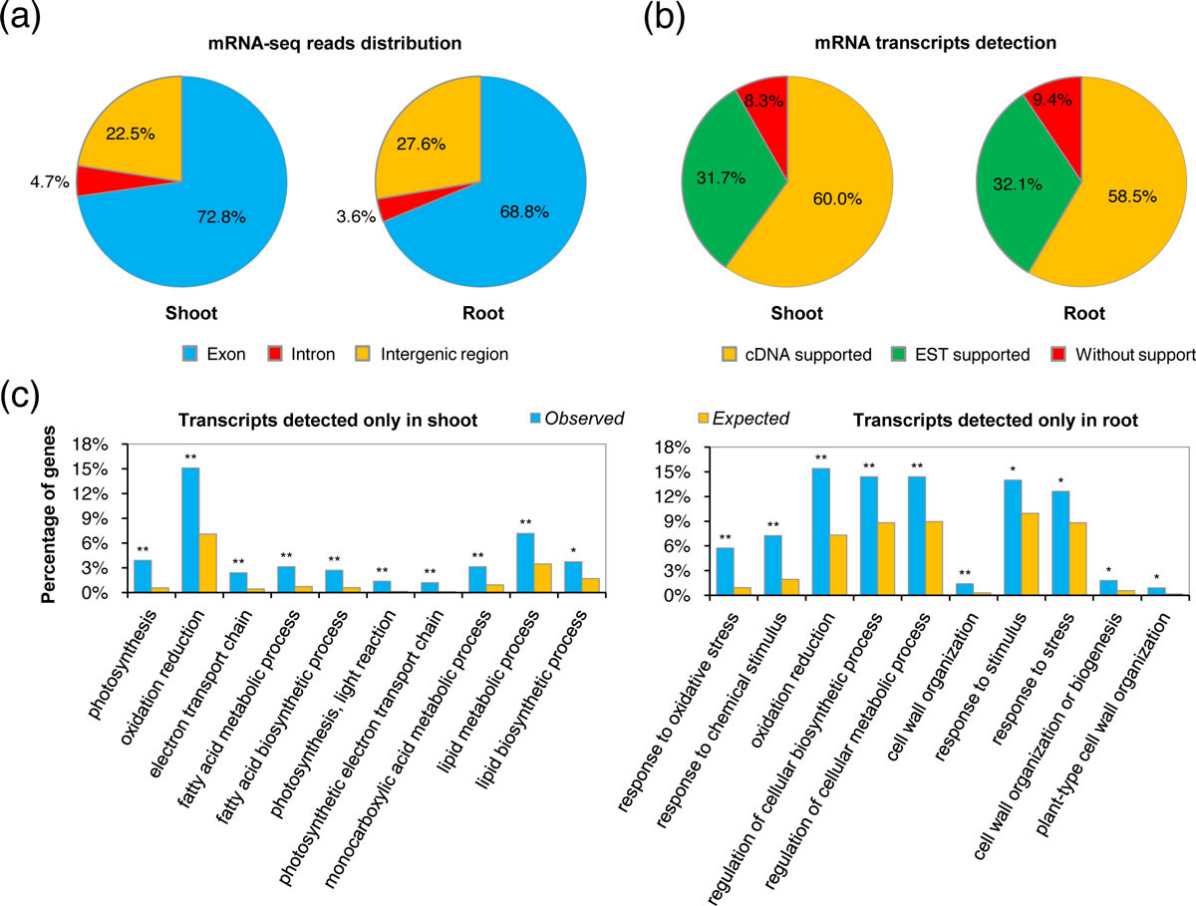
**Transcriptomic profiling in maize inbred lines and their reciprocal hybrids**. **(a) **Distribution of the mapped reads in the maize genome for mRNA sequencing (mRNA-seq) libraries from shoots and roots. For each organ, the mean percentages of both hybrids and parents are shown. **(b) **Proportion of mRNA transcripts identified in shoots and roots by mRNA-seq, according to gene annotations supported by expressed sequence tag (EST) or full-length cDNA data. For each organ, the mean percentages of both hybrids and parents are shown. **(c) **Functional categories of genes showing organ-specific expression. For each organ, only genes whose transcripts were detected in both hybrids and parents were included in the analysis. False-discovery rate adjusted *P*-values: *P<0.05 and **P<0.01, respectively.

To characterize the mRNA transcriptomes, we first investigated the distribution of reads across the annotated maize genome (release version 5b.60, filtered gene set). It was shown that, on average, 72.8% and 68.8% of the mRNA-seq reads in shoots and roots, respectively, were mapped to the annotated exons (Figure 1a). We used empirical cutoff values based on the comparison of mean read coverage between annotated exons and introns to assess the transcriptionally active genes in each mRNA-seq library (see Additional file 2, Figure S1). Comparisons were then made between transcriptionally active genes detected in our study and those from other experimental methods. For the 39,423 annotated genes in the maize genome, transcripts of 19,922 (50.5%) and 20,407 (51.8%) genes were detected in shoots and roots, respectively, of which 90.6% to 91.7% were supported by expressed sequence tags (ESTs) or full-length cDNAs (Figure 1b), indicating the reliability of our mRNA-seq data. Next, we investigated the Gene Ontology (GO) functional categories of genes whose transcripts were detected only in shoots (shoot-specific) or only in roots (root-specific). We found that shoot-specific genes (1,121 genes) were significantly enriched in the photosynthesis pathway, whereas root-specific genes (1,214 genes) were functionally enriched in the stress-response pathway (Figure 1c). Thus, we generated organ-specific transcriptomes of two maize inbred lines and their reciprocal hybrids.

**Figure 2:**
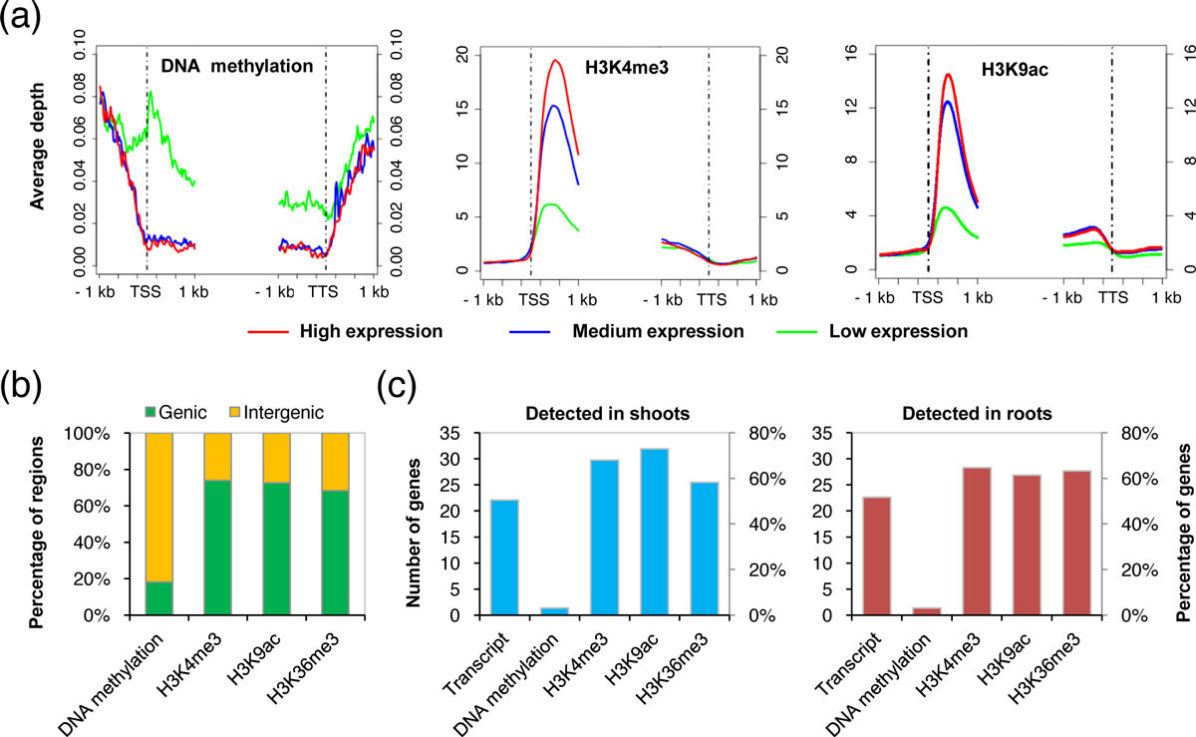
**Epigenomic profiling in maize inbred lines and their reciprocal hybrids**. **(a) **Distribution of DNA methylation, H3K4me3 and H3K9ac levels around transcription start site (TSS) and transcription termination site (TTS) of differentially expressed genes. Genes with detected transcripts were sorted according to their expression levels, and further divided into three groups (high, medium, and low expression levels, each with an equal number of genes). The mean read coverage of genes with epigenetic modifications was plotted (*y*-axis). **(b) **Frequencies of epigenetically modified regions in genic and intergenic regions of the maize genome. For each epigenetic mark, modified genomic regions identified using merged sequencing data from shoots and roots of both hybrids and parents were included in the analysis. **(c) **Number and percentage of genes identified with expression or epigenetic modifications. For each organ, only genes with detected transcripts or epigenetic modifications in both hybrids and parents were included.

To characterize the epigenomes, we first examined the mean read coverage of different epigenetic modifications relative to genes with differential expression. Our data showed that DNA methylation in genic regions correlated with transcriptional repression, whereas genic modifications of H3K4me3, H3K9ac and H3K36me3 were associated with active gene transcription (Figure 2a; see Additional file 2, Figure S2); these results were consistent with those of previous studies [7,8,14] and thus indicate the reliability of our epigenomic data. We also identified genomic regions associated with DNA methylation, and randomly selected nine regions and validated their *Mcr*BC-seq data by genomic bisulfite sequencing (see Additional file 2, Figure S3). Each histone modification was mapped to the corresponding genomic region using MACS software [42], and it was found that the majority (68.3 to 74.0%) of genomic regions with histone modifications (H3K4me3, H3K9ac, and H3K36me3) were associated with annotated genic regions (Figure 2b). By contrast, only 18.2% of genomic regions with DNA methylation were associated with annotated genic regions (Figure 2b).

Next, we analyzed the number and percentage of genes associated with expression or epigenetic modifications in shoots and roots. The levels of different epigenetic modifications on each gene were evaluated by directly counting the numbers of reads located in the genic region, and a threshold of read coverage defined by randomization (*P*<0.01) was used to identify genes with each modification. Generally, we obtained a similar number and percentage for genes with particular types of epigenetic modifications in shoots and roots (Figure 2c). Of 39,423 annotated genes in the maize genome, 22,696 to 28,786 (58.3% to 73.0%) and 24,248 to 25,532 (61.5% to 64.8%) genes in shoots and roots, respectively, contained histone modifications (H3K4me3, H3K9ac, and H3K36me3). However, only 1,243 (3.1%) and 1,276 (3.2%) genes contained DNA methylation in shoots and roots, respectively (Figure 2c). Together, we generated integrated maps of epigenomes and transcriptomes in shoots and roots of two maize inbred lines and their reciprocal hybrids (see Additional file 2, Figure S4 for a representative region on chromosome 1 showing the integrated maps).

### Patterns of variation in gene expression and epigenetic modifications between organs and between genotypes

To survey the global trends of transcriptional and epigenetic variation in different organs of maize hybrids, we performed genome-wide pairwise comparisons of gene expression and each epigenetic modification between organs and between genotypes. For each gene, the relative levels of its expression and epigenetic modifications were normalized to aligned reads per kilobase exon model per million mapped reads (RPKM) and aligned reads per kilobase genic (or genomic) region per million mapped reads (RPKM), respectively. To reduce the bias resulting from different sequencing coverages, only genes or genomic regions with detected transcripts or epigenetic modifications in both compared samples were included in a pairwise comparison. Genes or genomic regions showing significant discrepancy (*P*<0.05) in expression or epigenetic modifications between biological replicates were excluded from further analyses.

**Figure 3:**
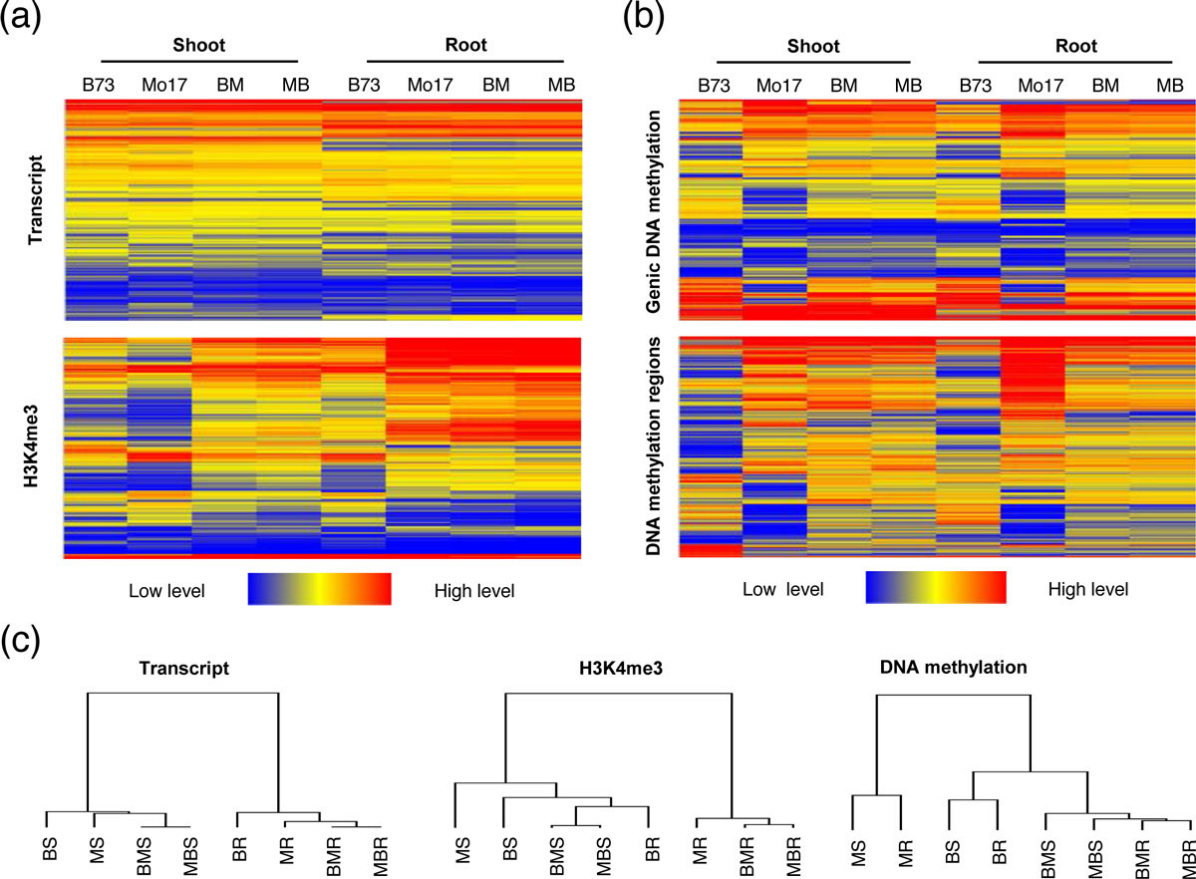
**Patterns of variations in transcriptomes and epigenomes between organs and between genotypes**. **(a,b) **Hierarchical clustering of transcription, histone modifications, and DNA-methylation levels in shoots and roots of maize hybrids and their parents. Only genes with significant differences in expression (*P*<0.001) or epigenetic modifications (*P*<0.01) in at least one pairwise comparison between organs or between genotypes were clustered using Cluster, version 3.0 (Ward's method, Euclidean distance) and visualized using Java TreeView, version 1.6.6r2. **(c) **Tree view of hierarchical clustering in (a) and (b). B, B73; M, Mo17; BM, B73 ´ Mo17; MB, Mo17 ´ B73; R, root; S, shoot.

To assess the transcriptional and epigenetic variation between organs and between genotypes, we performed hierarchical clustering, selecting genes with significant differences in expression or any epigenetic modifications in at least one organ or genotype. The clustering of expression data showed that the global patterns of transcriptomes were more distinguishable between shoot and root than between genotypes (Figure 3a), suggesting that variation in gene expression is more extensive between organs than between different genotypes (Figure 3c), which is consistent with a recent study [43]. The clustering of histone modification data showed that the global patterns of histone modifications (H3K4me3, H3K9ac, and H3K36me3) vary both between organs and between genotypes (Figure 3a,c; see Additional file 2, Figure S5). Moreover, the clustering of DNA-methylation data indicated that whereas DNA methylomes are similar between shoots and roots, they are distinct between hybrids and parents (Figure 3b).

Because the majority (81.8%) of methylated regions are located in intergenic regions in the maize genome (Figure 2b), we also performed hierarchical clustering using detected methylated regions. This analysis identified the same patterns of variation between organs and between genotypes as those found for genic DNA methylation (Figure 3b), thus indicating that patterns of variation in DNA methylation are affected more substantially by genotypes than by organs (Figure 3c). Consistent with this conclusion, a recent study using seedling shoots of the maize inbred lines B73 and Mo17 also suggested extensive variation in DNA methylation [20]. Moreover, further inspection of our data showed that genic DNA methylation in shoots and roots of hybrids exhibited B73-like patterns (Figure 3b), suggesting that parental difference in this repressive epigenetic mark is the main contributor to changed DNA methylation in hybrids, a phenomenon also reported in *Arabidopsis *hybrids [25].

### Histone modifications are associated with differential gene expression between organs and between hybrids and parents

Next, we explored the relationships between epigenomic variation and transcriptomic diversity between shoots and roots of the maize hybrids. Because the transcripts of most methylated genes (64.2% and 66.4% in shoots and roots, respectively) were undetectable, and because very few genes showed simultaneous variations in gene expression and DNA methylation between organs or between hybrids and parents, genic DNA methylation was excluded from further analysis in this part of the study.

**Figure 4:**
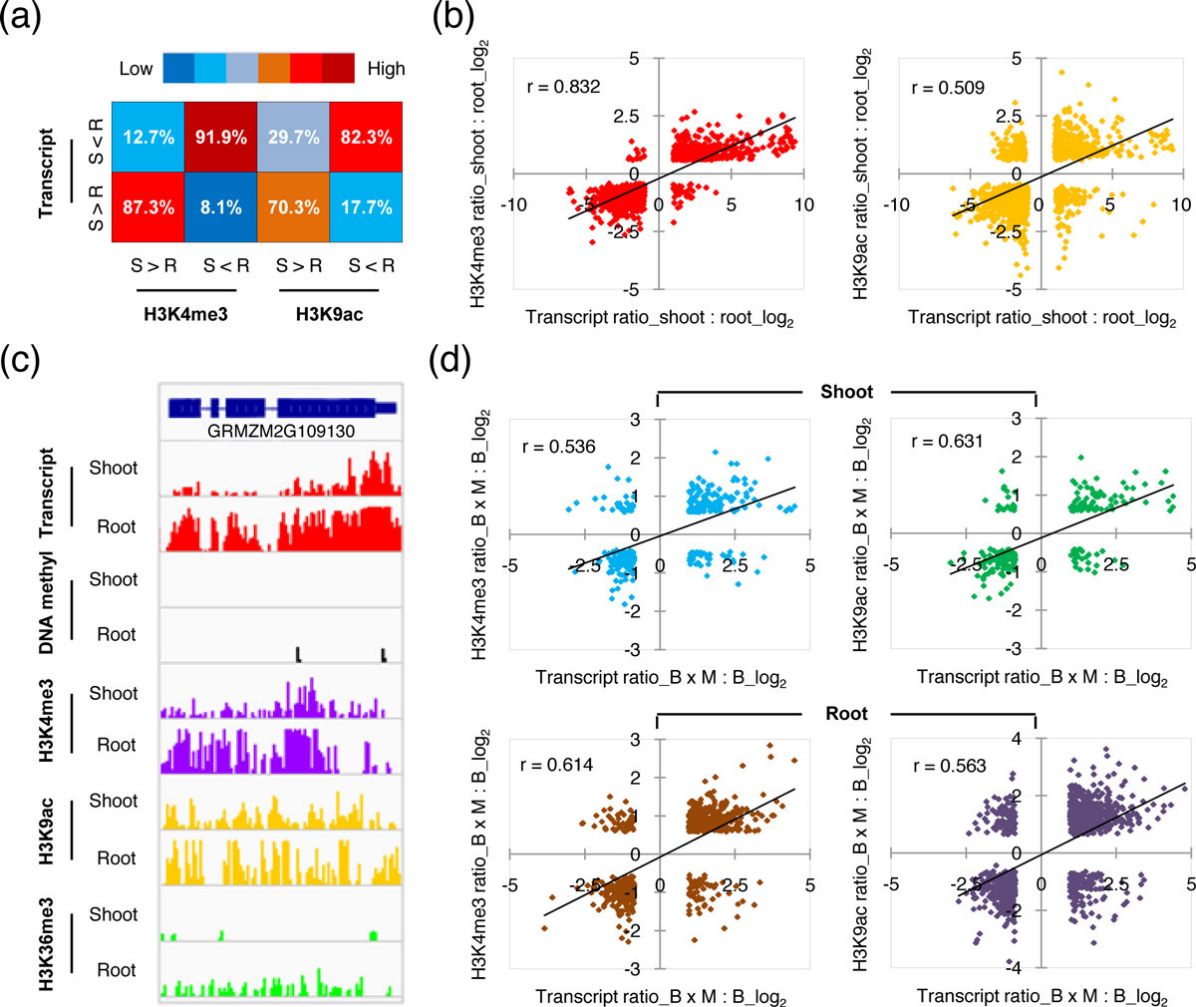
**Relationships of variations in gene expression and histone modifications between organs and between genotypes**. **(a) **Frequencies of concurrence between variations in gene expression and histone modifications between shoots and roots. R, root; S, shoot. Numbers indicate the percentage of differentially modified genes that were also differentially expressed. **(b) **Correlations between differential gene expression (*P*<0.01 and fold change >2.0) and histone modifications (*P*<0.01 and fold change >1.5) between shoots and roots. **(c) **A typical maize gene showing differential gene expression and epigenetic modifications in shoots and roots. DNA methyl, DNA methylation. **(d) **Correlations between differential gene expression (*P*<0.01 and fold change >2.0) and histone modifications (*P*<0.01 and fold change >1.5) between hybrids and parents.

To investigate the relationships between variations in histone modifications and gene expression between organs, we first counted the frequencies of concurrence between differential histone modifications and gene expression (Figure 4a). We found a high level of concurrence between differences in histone modifications and in gene expression (Figure 4a). For example, for those genes with higher levels of H3K4me3 in shoots, 87.3% also had higher levels of expression in shoots. We further selected the genes with significant differences in both transcripts (*P*<0.001 and fold change >2.0) and epigenetic modifications (*P*<0.01 and fold change >1.5) between shoots and roots, so as to quantitatively examine the correlation between differences in histone modifications and in gene expression between organs. We found that histone modifications positively correlated with differential gene expression, especially for H3K4me3 (Pearson correlation = 0.832, *P*<0.01) (Figure 4b; see Additional file 2, Figure S6). These observations indicate that histone modifications are associated with differential gene expression between shoots and roots in maize (Figure 4c). We also investigated the relationship between variations in histone modifications and in gene expression between hybrids and parents, and found positive correlations between differences in gene expression and in these three histone modifications (Figure 4d; see Additional file 2, Figure S6). These results indicate that histone modifications are associated with variation in gene expression in both shoots and roots of maize hybrids.

### Genes that are upregulated in shoots and roots of hybrids are significantly enriched in the nucleosome assembly pathway

To exploit the biological implication of differential gene expression or epigenetic modifications in different organs of maize hybrids, we examined the functional categories of genes in each pattern of variation. The modes of gene action in hybrids are classified as additive and non-additive, and the latter is further subdivided into upregulation or downregulation relative to the mid-parent value (MPV). To ensure the reliability of the data, we used only sequencing reads that mapped uniquely to the genome of both B73 and Mo17, and used a significance level of *P*<0.001 to identify the differentially expressed or modified genes between hybrids and parents. To exclude discrepancies between reciprocal hybrids, only genes showing the same pattern of variation in reciprocal hybrids were included in the analyses. Moreover, to reduce the bias resulting from different sequencing coverages, and to make the results comparable between organs, only genes whose transcripts or epigenetic modifications were detected in both shoots and roots of both hybrids and parents were included in the functional analysis. These genes were then subjected to GO analysis using agriGO software [44]. With respect to each epigenetic mark, no biological pathway was significantly enriched in genes showing additive or non-additive epigenetic modifications in hybrids.

**Figure 5:**
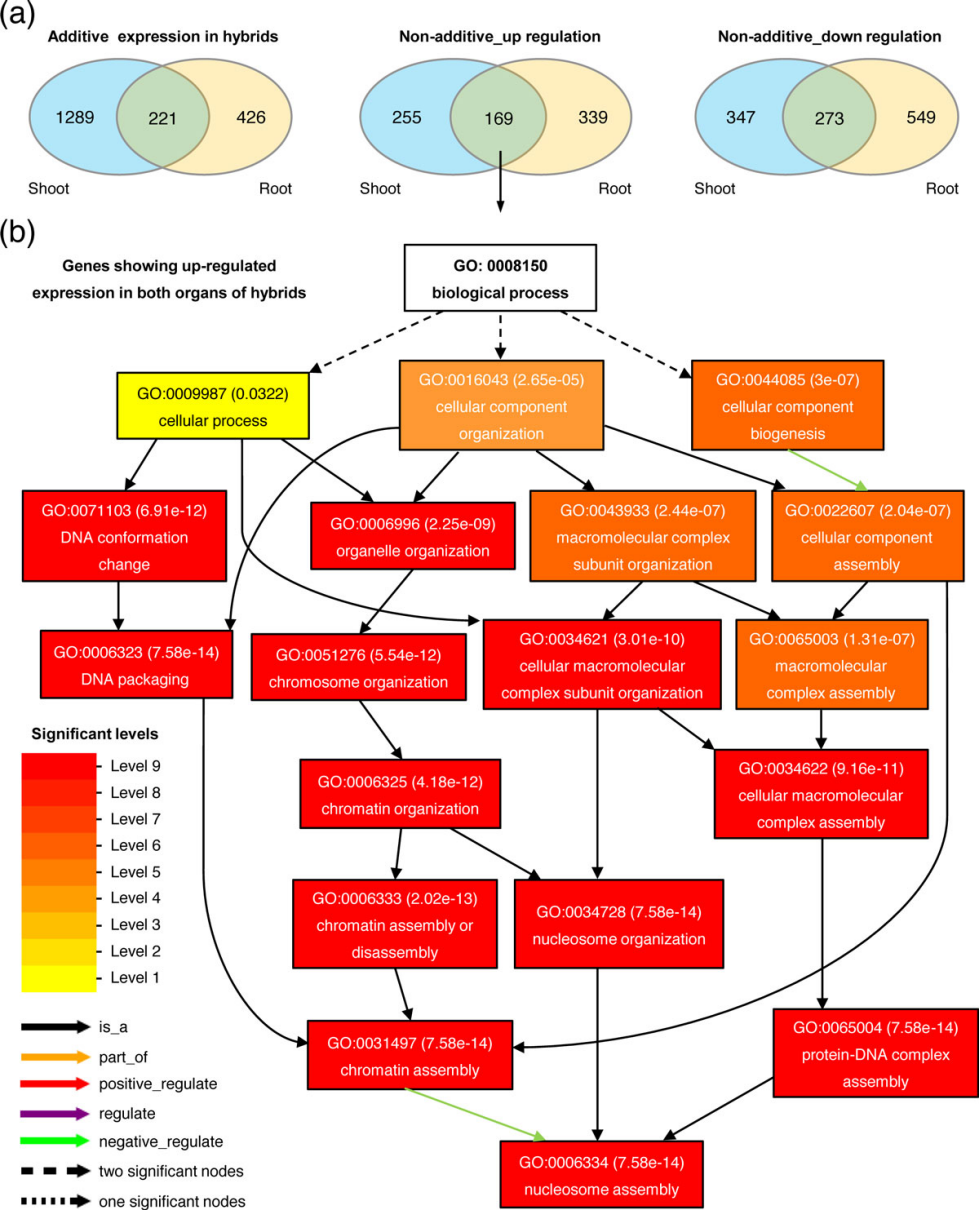
**Variations in gene expression in shoots and roots of maize hybrids**. **(a) **Identification of genes showing additive or non-additive expression in shoots and roots of maize hybrids. Only genes showing the same pattern of expression variations between reciprocal hybrids were included. **(b) **Functional categories of genes upregulated in both shoots and roots of hybrids. The biological process with false-discovery rate adjusted *P*-value <0.01 is shown. GO, Gene Ontology.

In total, 1,510 (false-discovery rate (FDR) = 0.0017) and 647 (FDR = 0.012) genes exhibiting additive expression were identified in shoots and roots of reciprocal hybrids, respectively, of which, 221 genes had additive expression in both organs (Figure 5a; see Additional file 3, Table S2). Functional analysis showed that no biological pathway was enriched in genes additively expressed in either shoots or roots. In addition, we identified 1,044 (FDR = 0.0075) and 1,330 (FDR = 0.0059) genes showing non-additive expression in shoots and roots of reciprocal hybrids, respectively. Of these, 424 and 508 genes showed upregulation, whereas 620 and 822 showed downregulation in shoots and roots of reciprocal hybrids, respectively (Figure 5a; see Additional file 3 Table S2). Functional analysis showed that genes upregulated in shoots or roots of hybrids were significantly enriched in the nucleosome assembly pathway (for shoots, *P *= 3.4 ´ 10^-12^, FDR = 6.2 ´ 10^-10^; for roots, *P *= 1.8 ´ 10^-13^, FDR = 3.9 ´ 10^-11^) (see Additional file 2, Figure S7). By contrast, no biological pathway was enriched for genes downregulated in either shoots or roots of reciprocal hybrids.

We further analyzed the biological functions of genes showing organ-specific non-additive expression, and of genes showing the same pattern of non-additive expression in both organs. Of 255 and 339 genes upregulated only in shoots or roots of reciprocal hybrids, respectively, no biological pathway was significantly enriched. However, the nucleosome assembly pathway was significantly enriched for genes upregulated in both organs (*P*= 6.8 ´ 10^-16^, FDR = 7.62 ´ 10^-14^) (Figure 5b). In addition, no biological pathway was enriched for genes showing either organ-specific downregulation, or in genes downregulated in both organs. These data suggest that upregulation of gene expression may be associated with the nucleosome assembly pathway, and that this association may be a common regulatory mechanism in both shoots and roots of maize hybrids.

### Parental alleles contribute similarly to biased expression in both organs of reciprocal hybrids

**Figure 6:**
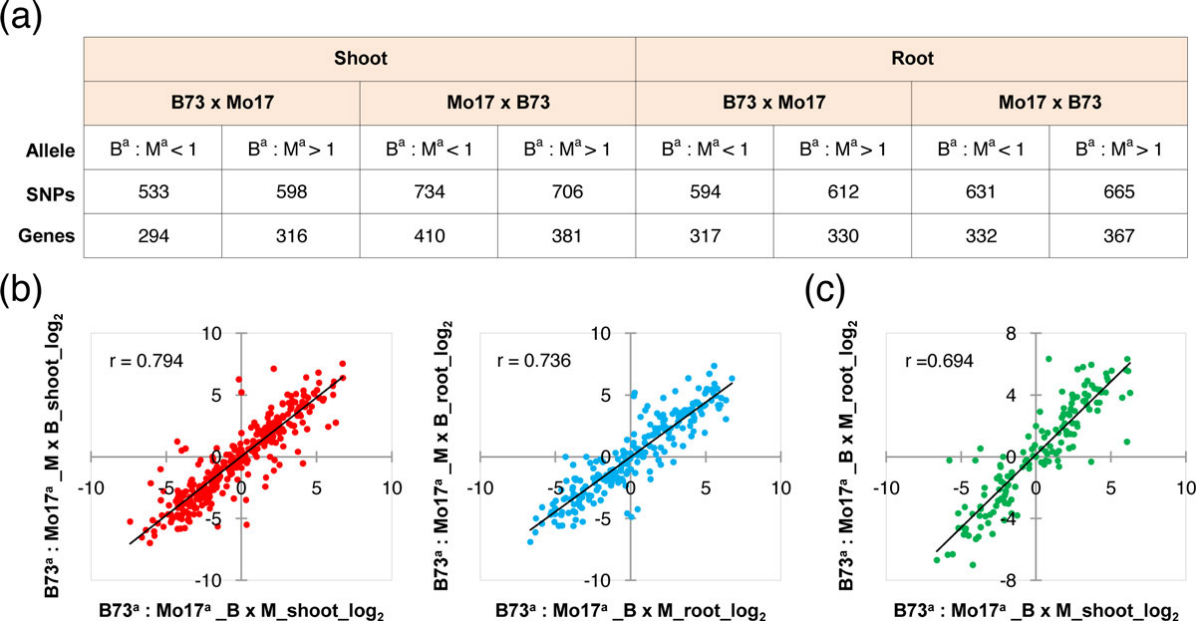
**Allelic expression bias in shoots and roots of reciprocal hybrids**. **(a) **Detection of allelic expression bias in hybrids with a *P*-value cutoff of 0.05. B^a^, B73 allele; M^a^, Mo17 allele. **(b) **Correlation of allelic expression bias between reciprocal hybrids. **(c) **Correlation of allelic expression bias between shoots and roots of B73 ´ Mo17.

Previous studies reported that parental alleles show biased expression in different organs of maize hybrids [45,46]. To better understand how parental alleles contribute to differential gene expression or epigenetic modifications in different organs of maize hybrids, we performed allelic bias analysis in hybrids using single-nucleotide polymorphisms (SNPs) identified by comparing our transcriptomic and epigenomic sequencing reads at each base pair of 20,850 homologous genes between parental lines B73 and Mo17. Allele-specific sequencing reads discriminated by the identified SNPs were used to evaluate allelic expression or epigenetic bias in hybrids using a binomial test, with the null hypothesis that two parental alleles are uniformly expressed or modified in the hybrids [8]. To reduce the effects of the divergence of genomic sequences between two parental lines, only the sequencing reads mapping uniquely to the genomes of both B73 and Mo17 were included in the analysis. At *P*<0.05, 533 to 734 SNPs (294 to 410 genes) and 594 to 665 SNPs (317 to 367 genes) that showed biased allelic expression were identified in shoots and roots of reciprocal hybrids, respectively (Figure 6a). However, very few SNPs were identified that could discriminate allelic bias in epigenetic modifications, therefore these were excluded from further analyses. Discrimination of the differential allelic expression based on the direction of allelic bias in hybrids showed no obvious bias toward either B73 or Mo17 (Figure 6a), suggesting that in both shoots and roots of maize, the parental genomes contribute equally to the activity of the transcriptomes in hybrids.

Our previous study in rice showed that there was no significant parent-of-origin effect for the action of parental alleles in hybrids [8]. In the current study, we also examined whether this conclusion is true or not in maize hybrids. Of 354 and 249 genes with biased expression in shoots and roots of B73 ´ Mo17 and Mo17 ´ B73, respectively, 333 (94.1%) and 222 (89.2%) exhibited the same direction of biased expression in shoots and roots of both hybrids, respectively. Further quantitative analysis showed that in both shoots and roots, there is a strong positive correlation of differential allelic expression between reciprocal hybrids (Figure 6b). These data suggest that, similar to rice, there is no obvious parent-of-origin effect in shoots and roots of maize hybrids that is responsible for the allelic bias.

To investigate whether there are organ-specific effects of allelic expression in maize hybrids, we identified genes showing biased expression in both shoots and roots of hybrids, and compared the direction of biased expression between organs. We found that, of 170 genes with biased expression in both shoots and roots of B73 ´ Mo17, 146 (85.9%) exhibited the same direction of biased expression in both organs of hybrids. Similarly, of 284 genes with biased expression in both organs of Mo17 ´ B73, 261 (91.9%) exhibited the same direction of biased expression. Further quantitative analysis identified a high positive correlation of differential allelic expression between shoots and roots of hybrids (Figure 6c; see Additional file 2, Figure S8), suggesting that the regulatory mechanisms of allelic bias in these organs may be the same.

### Small interfering RNAs of 22 and 24 nucleotides in length are derived from distinct transposable elements and are differentially accumulated between hybrids and parents

We also examined sRNA transcriptomes in maize hybrids and their parental lines using sRNA-seq. After removing the adapter sequences and sequencing reads associated with rRNAs, tRNAs, and small nuclear and nucleolar RNAs, sRNA-seq reads were aligned to the reference genome of the maize inbred line B73 (ZmB73_RefGen_v2) [31]. We obtained only a small number of mapped reads from two sRNA-seq libraries (derived from shoots of Mo17 ´ B73 and roots of B73 ´ Mo17), therefore these two libraries were excluded from further analyses.

The sRNA-seq reads corresponding to the precursors of each known maize microRNA (miRNA) were used to characterize miRNA expression in maize hybrids and their parental lines (see Additional file 3, Table S3). The remaining sRNA reads from all libraries were pooled and used to identify 21 nt, 22 nt, and 24 nt siRNA clusters by clustering adjacent sRNA reads of 21 nt, 22 nt, and 24 nt in length, respectively. An siRNA cluster was defined as a region containing a minimum of six sRNA reads, each separated from the nearest neighbor by a maximum of 200 nt. When associated with gene annotations, the identified 21 nt, 22 nt, and 24 nt siRNA clusters were all enriched in a 2 kb area upstream or downstream of the transcribed gene regions (see Additional file 2, Figure S9), similar to the observations in rice [8] and *Arabidopsis *[47], although with different total abundance.

**Figure 7:**
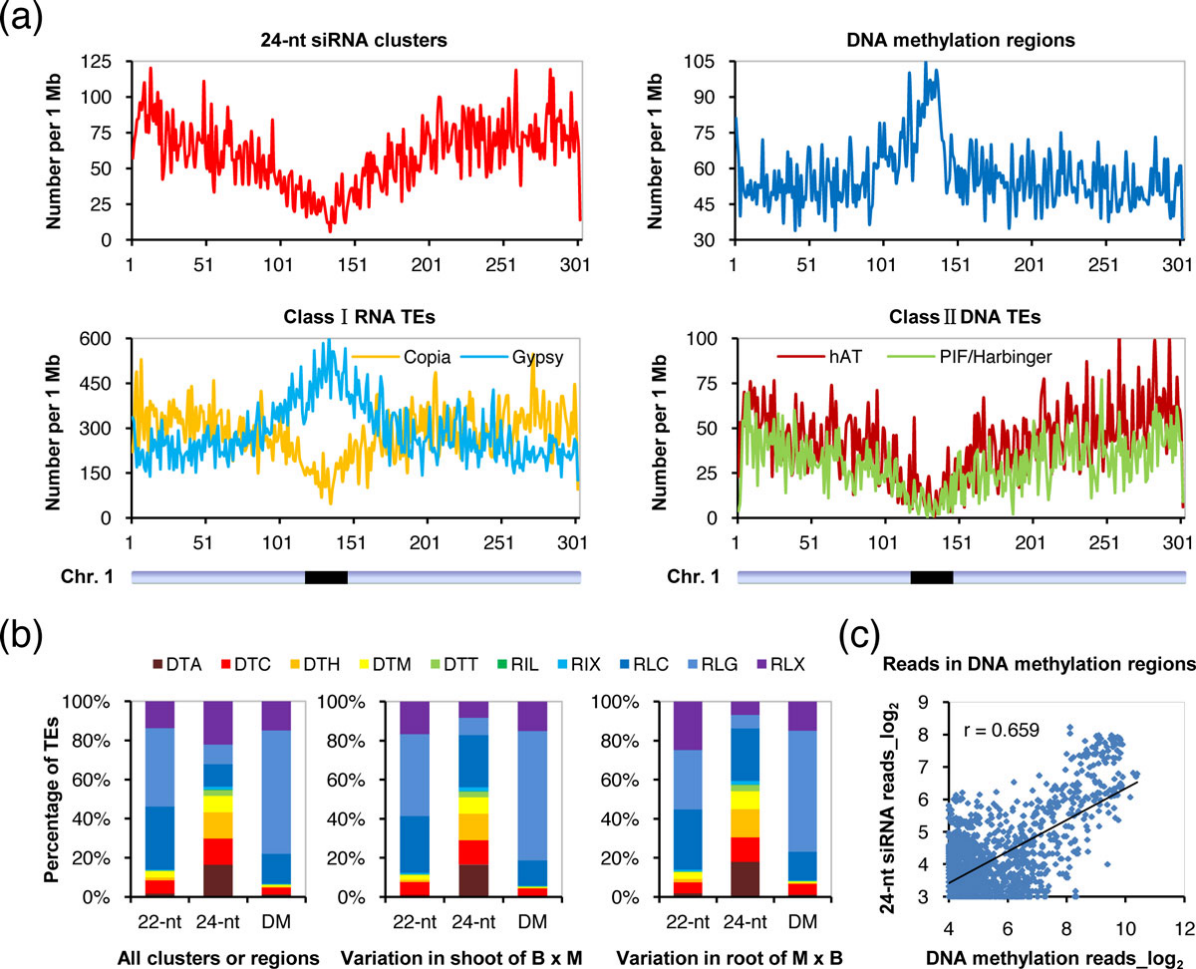
**Relationships of small interfering RNAs (siRNAs), DNA methylation, and transposable elements (TEs) and their variations in maize hybrids**. **(a) **Distribution of 24 nt siRNA clusters, DNA methylation, and main TE classes on maize chromosome 1. **(b) **Overlap of 22 nt and 24 nt siRNA clusters and methylated DNA regions with distinct TE classes in the maize genome. B, B73; M, Mo17; DTA, *hAT*; DTC, *CACTA*; DTH, *PIF/Harbinger*; DTM, *Mutator*; DTT, *Tc1/Mariner*; RIL, *LINE*; RIX, Unknown *LINE*; RLC, *Copia*; RLG, *Gypsy*; RLX, Unknown *LTR*. **(c) **Correlation between 24 nt siRNAs and DNA-methylation levels at the same genomic loci.

We then investigated the distribution of genomic sequences covered by siRNA clusters across the maize genome, and found a wide distribution of 22 nt siRNAs along each chromosome, with no obvious enrichment in euchromatic or heterochromatic regions (see Additional file 2, Figure S10). Unexpectedly, the 24 nt siRNAs showed low abundance in pericentromeric regions but were highly enriched in euchromatic regions (Figure 7a). The distribution pattern of 24 nt siRNAs along chromosomes contrasted with that of DNA methylation, which showed strong enrichment in heterochromatic regions (Figure 7a). However, the 21 nt siRNAs showed a weak bias toward the euchromatic regions (see Additional file 2, Figure S10). Because the endogenous siRNAs identified to date tend to be produced from repetitive sequences including TEs [13], and nearly 85% of the maize genome is composed of TEs [31], we further explored the relationship between different siRNA species and various TE classes in the maize genome. As reported previously [31], among class I RNA TEs, *Copia *elements are enriched in euchromatic regions, whereas *Gypsy *elements are highly enriched in heterochromatic regions, similar to the distribution pattern of DNA methylation (Figure 7a). Among class II DNA TEs, *CACTA *elements show unbiased distribution along chromosomes, whereas *hAT*, *PIF/Harbinger*, and all other elements are enriched in euchromatic regions, similar to the distribution pattern of 24 nt siRNAs (Figure 7a) [31].

To investigate how siRNAs correlate with DNA methylation and TEs in maize, we analyzed the co-occurrence of TEs with siRNA clusters or DNA methylation across the maize genome. We calculated the number of siRNA clusters or methylated regions with 50% minimum length overlapping with different classes of annotated TEs, and compared the proportion of each class of TEs relative to that in the whole genome. We found that among the 21 nt siRNA clusters co-occurring with TEs, no TE classes were obviously enriched. However, among the 22 nt siRNA clusters co-occurring with TEs, the *Copia *and *Gypsy *elements of class I RNA TEs were significantly over-represented (32.2% and 40.1%, respectively; *P*<0.001, c^2 ^test), whereas the *hAT*, *CACTA*, and *PIF/Harbinger *elements of class II DNA TEs were significantly under-represented (1.8%, 6.7% and 1.6% respectively; *P*<0.001, c^2 ^test) (Figure 7b). By contrast, among the 24 nt siRNA clusters co-occurring with TEs, *Copia *and *Gypsy *were significantly under-represented (11.5% and 9.9%, respectively; *P*<0.001, c^2 ^test), whereas *hAT*, *CACTA*, and *PIF/Harbinger *were all significantly over-represented (16.5%, 13.4% and 13.3%, respectively; *P*<0.001, c^2 ^test) (Figure 7b). These data show that 22 nt siRNAs tend to be produced from *Copia *and *Gypsy *elements of class I RNA TEs, whereas 24 nt siRNAs tend to be produced from *hAT*, *CACTA*, and *PIF/Harbinger *elements of class II DNA TEs, suggesting that different siRNA species are derived from distinct TE classes.

We also found that among methylated DNA regions co-occurring with TEs, the *Gypsy *element of class I RNA TEs was significantly over-represented (63.0%; *P*<0.001, c^2 ^test), whereas *Copia*, *hAT*, *CACTA*, and *PIF/Harbinger *elements were all under-represented (Figure 7b), suggesting that *Gypsy *elements are highly methylated in the maize genome. Similar results were also obtained when we analyzed the siRNA clusters or methylated DNA regions that showed variation in shoots or roots of hybrids relative to their parents. These data suggest that in both organs, variations in siRNA activity in hybrids for *hAT*, *CACTA*, and *PIF/Harbinger *elements of class II DNA TEs are primarily driven by 24 nt siRNAs, whereas the differences in siRNA activity between hybrids and patents for *Copia *and *Gypsy *elements of class I RNA TEs are primarily driven by 22 nt siRNAs (Figure 7b).

## Discussion

How the combined genomes of parents are regulated in hybrids so as to generate significant differences in genome activities between hybrids and parents is a fundamental biological question. Recent studies suggest that such differences could be ascribed to epigenetic variations [8,24,25,48]. In addition, similarities and differences in gene expression in distinct organs of maize hybrids, such as seedling shoots [37,38] and roots [39,40] have also been reported. In this study, we investigated the global variation in transcriptomes and epigenomes in shoots and roots of the B73 and Mo17 inbred lines and their reciprocal hybrids. Our data showed that for each examined epigenetic component in the maize genome, there were no obvious differences in global distribution patterns between organs and between hybrids and parents. However, expression of specific genes or epigenetic modifications at specific genomic loci exhibited significant quantitative variation between hybrids and parents, and between different organs. Our data showed that the patterns of variation in gene expression and each epigenetic modification were distinct. Although many genes showed variation in expression in hybrids, the global patterns of gene expression showed more extensive variation between organs than between hybrids and parents (Figures 3a,c). Conversely, variation in DNA-methylation patterns was more extensive between genotypes than between organs (Figures 3b,c), suggesting a limited contribution of DNA methylation to maize development. Because only a small number of genes are identified with DNA methylation in their transcribed regions (Figure 2b) and very few genes showed simultaneous variation in gene expression and DNA methylation between hybrids and parents, the extensive variation in DNA methylation in maize hybrids were mainly associated with the activity of TEs (especially the *Gypsy *elements) and therefore would be expected to affect the genomic stability of hybrids (Figure 7b). Moreover, we found that histone modifications varied extensively both between organs and between genotypes (Figures 3a,c; see Additional file 2, Figure S5), and were associated with differential gene expression between organs and between hybrids and parents (Figure 4; see Additional file 2, Figure S6). These results suggest that histone modifications, which are strongly associated with transcribed regions (Figure 2b), play important roles in expression divergence both between organs and between genotypes. By contrast, DNA methylation, which is largely associated with intergenic regions (Figure 2b), may play specific roles in driving the variation in stability and activity of the hybrid genomes by altering the chromatin states.

Many studies have attempted to identify specific gene sets or pathways responsible for hybrid vigor in plants by investigating differential gene expression between hybrids and their parental inbred lines [49,50]. In the current study, we found that no biological pathway was enriched for genes showing additive or downregulated expression in shoots and roots of reciprocal hybrids; however, genes upregulated in shoots and roots of hybrids were significantly enriched in the nucleosome assembly pathway (Figure 5b; see Additional file 2, Figure S7). As a fundamental biological process required for chromosome replication and maintenance, nucleosome assembly is closely coupled with cell division, and is strongly upregulated during the S-phase of the cell cycle. In addition, it also contributes to the inheritance of chromatin states, and influences the regulation of gene activity and other processes that act on DNA [51,52]. Because the nucleosome assembly pathway was enriched only for genes upregulated in both organs of hybrids, and because no biological pathway was enriched for genes showing organ-specific expression in hybrids, our data suggest that the enrichment of the nucleosome assembly pathway is likely to be one of the common molecular events in both shoots and roots of maize hybrids. A recent study showed that a dramatic reduction in 24 nt sRNAs strongly affected the expression of genes responsible for chromatin modifications [53], thus the enrichment of the nucleosome assembly pathway shown in the current study might be a molecular response to the large-scale changes in sRNA profiles and the RdDM pathway. However, whether this pathway is associated with hybrid vigor awaits further investigation. By contrast, no biological pathway was found to be enriched for genes showing downregulated expression in both organs of reciprocal hybrids. This may be a reflection of the fact that downregulated genes, and upregulated genes other than those involved in the nucleosome assembly pathway, are associated with various biological functions resulting from genome-wide genetic variations in hybrids.

Several recent studies have explored the differences in sRNA transcriptomes between hybrids and parents [8,21,23-30]. In the current study, we found that much more siRNA clusters were downregulated (11,558) than upregulated (2,911) in both organs of maize hybrids, consistent with the observation that siRNAs tend to be downregulated in hybrids [8,21,25,28]. Because siRNAs are involved in transcriptional silencing of TEs through the RdDM pathway [54], we therefore investigated the relationships between siRNAs, DNA methylation, and different TE classes in the maize genome. A recent study showed that 21 and 22 nt siRNAs are derived from distinct retrotransposon families, and are differentially accumulated between the maize inbred lines B73 and Mo17 and their hybrids [28]. Another study also identified a distinct size preference of sRNAs resulting from different TE families, and suggested the 22 nt sRNAs as a major component in the silencing of most TE families in soybean [15]. Our data also show that different TE classes tend to produce distinct siRNA species (Figure 7b). This observation suggests divergent effects of different TEs on chromatin states, as reported in a recent study, which reported that there are family-specific attributes for the effects of TEs on neighboring chromatin [55].

In addition, we found that whereas DNA methylation was found to be highly enriched in heterochromatic regions, siRNA clusters were not obviously enriched (for 22 nt siRNAs), or even devoid (for 21 and 24 nt siRNAs) in these regions (Figure 7a; see Additional file 2, Figure S10). However, a positive correlation between 22 nt and 24 nt siRNAs and DNA-methylation levels at the same genomic loci were also seen (Figure 7c; see Additional file 2, Figure S11). A possible explanation for these observations may be that both siRNA-dependent and siRNA-independent pathways are responsible for methylation of TEs in the maize genome, or alternatively, TE sequences in heterochromatic regions acquire DNA methylation through spreading from adjacent siRNA-targeted regions [56].

## Conclusions

In this study, we investigated the conservation and divergence of transcriptomic and epigenomic variations in shoots and roots of two maize inbred lines and their reciprocal hybrids. The global distribution patterns of epigenetic components between parents and hybrids contained extensive variations in the levels of DNA methylation, histone modifications, and siRNA transcription, which are conserved between shoots and roots. These diverse epigenetic variations potentially make important contributions to altered genome activity in different organs of hybrids compared with their parents by modulating chromatin states so as to accommodate hybridization. Confirmation of these results awaits further studies exploring integrated transcriptomic and epigenomic profiling with more extensive sequencing in more organs of more hybrids.

## Materials and methods

### Plant materials and growth conditions

The inbred lines B73 and Mo17 of maize (*Zea mays*) and their reciprocal F_1 _hybrids (B73 ´ Mo17 and Mo17 ´ B73) were used in this study. Seeds were grown in soil under controlled environmental conditions (15 hours of light at 25°C, and 9 hours dark at 20°C) in a growth chamber. After 14 days, seedling shoots and roots were harvested, then, frozen in liquid nitrogen and stored at -80°C for isolation of DNA and total RNA, or processed directly for ChIP assays after harvesting.

### Sample preparation and sequencing library construction

Three independent biological replicates, each consisting of three pooled shoots or roots of the hybrids and parental lines, were used for constructing mRNA-seq, *Mcr*BC-seq, ChIP-seq, and sRNA-seq libraries, and each library was sequenced in a single lane as described previously [3,7,8]. Briefly, total RNAs were isolated using TRIzol reagent (Invitrogen Corp., Carlsbad, CA, USA) and treated with RNase-free DNase I (New England Biolabs, Ipswich, MA, USA) to remove any contaminating genomic DNA. mRNA extraction was performed using Dynabeads oligo(dT) (Dynal; Invitrogen Corp.). Double-stranded cDNAs were synthesized using reverse transcriptase (Superscript II; Invitrogen Corp.) and random hexamer primers. The cDNAs were then fragmented by nebulization, and the standard Illumina protocol was followed thereafter to create the mRNA-seq libraries. Genomic DNAs were isolated using a commercial kit (DNeasy Plant Maxi Kit; Qiagen Inc., Valencia, CA, USA). Isolated genomic DNAs were then digested with *Mcr*BC (New England Biolabs) followed by gel purification to enrich methylated genomic DNAs. The *Mcr*BC-seq libraries were generated using the standard Illumina protocol. The ChIP-seq libraries were generated by immunoprecipitating chromatin with antibodies against H3K4me3 (Abcam, Cambridge, UK), H3K9ac (Upstate Biotechnology, Lake Placid, NY, USA), or H3K36me3 (Abcam), as described previously [57]. The eluted ChIP DNAs from the three ChIP reactions were pooled to generate ChIP-seq libraries for Illumina sequencing, following the manufacturer's protocol. sRNAs were gel-purified from total RNAs, and were subsequently ligated with 3' and 5' adapters, followed by reverse transcription using a 3' reverse transcriptase primer. The cDNAs were then amplified by PCR using primers specific to sRNAs [58]. After gel purification, the sRNA-seq libraries were subjected to Illumina sequencing following the manufacturer's protocol.

The original datasets have become public in the NIH GEO database under the accession [GEO: GSE43142].

### Data processing and analyses

For the methods used in the analysis of data from mRNA-seq, *Mcr*BC-seq, ChIP-seq and sRNA-seq, see Additional file 4.

## Abbreviations

cDNA: Complementary DNA; ChIP-seq: Chromatin immunoprecipitation sequencing; EST: Expressed sequence tag; F_1_: First filial generation; FDR: False-discovery rate; gDNA: Genomic DNA; GO: Gene Ontology; H3K36me3: Histone H3 tri-methylated at lysine 36; H3K4me3: Histone H3 tri-methylated at lysine 4; H3K9ac: Histone H3 acetylated at lysine 9; MACS: Model-based analysis of ChIP-seq; *Mcr*BC-seq: *Mcr*BC sequencing; miRNA: MicroRNA; MPV: Mid-parent value; mRNA: Messenger RNA; mRNA-seq: mRNA sequencing; PCR: Polymerase chain reaction; RdDM: RNA-directed DNA methylation; RPKM: Aligned reads per kilobase exon model (or genic region: or genomic region) per million mapped reads; siRNA: Small interfering RNA; SNP: Single-nucleotide polymorphism; sRNA: Small RNA; sRNA-seq: sRNA sequencing; TE: Transposable element; TSS: Transcription start site; TTS: Transcription termination site.

## Competing interests

The authors declare that they have no competing interests.

## Authors' contributions

XWD conceived of the project. XWD, XL and GH designed the experiments. GH, XL, MY, LL and YQ performed the experiments. GH, BC, XW, HH and LL conducted data analyses. GH, JL, LL, XW and XWD prepared the article. All authors read and approved the final manuscript.

## Supplementary Material

Additional file 1 **Table S1: **Summary of total reads obtained from all sequencing libraries. Click here for file

Additional file 2 **Figures S1 to 11**. Figure S1: Mean levels of exons and introns in shoots and roots of reciprocal hybrids. Figure S2: Distribution of H3K36me3 levels within and around differentially expressed genes. Figure S3: Experimental validation of methylated DNA regions by genomic bisulfite sequencing. Figure S4: A representative genomic region on maize chromosome 1 showing integrated maps of transcription and epigenetic modifications. Figure S5: Tree view of hierarchical clustering of H3K9ac and H3K36me3 levels. Figure S6: Relationships of variations in H3K36me3 and gene expression between organs and between genotypes. Figure S7: Functional categories of genes upregulated in shoots and roots of hybrids. Figure S8: Correlation of allelic expression bias between shoots and roots of Mo17 ´ B73. Figure S9: Coverage of 21 nt, 22 nt, and 24 nt siRNA clusters in and around protein-coding genes. Figure S10: Distribution of 21 nt and 22 nt siRNA clusters on maize chromosome 1. Figure S11: Correlation between 22 nt siRNAs and DNA-methylation levels at the same genomic loci. Click here for file

Additional file 3 **Tables S2 and 3**. Table S2: List of genes showing expression variation in shoots and roots of maize hybrids. Table S3: Number of small RNA reads associated with known maize microRNAs. Click here for file

Additional file 4 **Supplemental methods**. Details of data processing and analyses. Click here for file
