# Supplementary material for: Conservation and divergence of transcriptomic and epigenomic variation in maize hybrids
Source: Genome Biol. 2013 Jun 12;14(6):R57. doi: 10.1186/gb-2013-14-6-r57 (PMC3707063; doi:10.1186/gb-2013-14-6-r57)
Supplement: Additional file 1 — Table S1: Summary of total reads obtained from all sequencing libraries. [file gb-2013-14-6-r57-S1.PDF]

Table S1. Summary of total reads obtained from all sequencing libraries

	B73	Mo17	B73 x Mo17	Mo17 x B73
**Transcript**				
Shoot	24,256,798	14,327,415	17,671,936	17,232,066
Root	22,428,111	15,634,737	15,631,331	19,008,589
**DNA methylation**				
Shoot	11,978,618	9,045,055	21,849,136	18,271,125
Root	12,782,516	8,095,044	19,945,254	21,072,218
**H3K4me3**				
Shoot	6,434,140	8,102,337	11,801,772	11,349,156
Root	7,570,065	5,878,785	7,282,921	10,237,027
**H3K9ac**				
Shoot	8,873,929	8,627,226	15,468,267	14,511,465
Root	6,229,907	7,569,282	11,397,416	10,055,300
**H3K36me3**				
Shoot	8,170,906	7,780,725	11,790,825	13,247,479
Root	7,798,190	9,417,630	12,207,502	12,686,266
**small RNA**[1]				
Shoot	4,468,838	5,118,485	10,104,381	NA
Root	3,050,450	7,775,771	NA	10,372,967

The numbers of reads matched to the genome of maize inbred line B73 are indicated.

[1]Reads are calculated after the removal of rRNAs, tRNAs, snRNAs and snoRNAs.

NA, not available.
