# Supplementary material for: Conservation and divergence of transcriptomic and epigenomic variation in maize hybrids
Source: Genome Biol. 2013 Jun 12;14(6):R57. doi: 10.1186/gb-2013-14-6-r57 (PMC3707063; doi:10.1186/gb-2013-14-6-r57)
Supplement: Additional file 2 — Figures S1 to 11. Figure S1: Mean levels of exons and introns in shoots and roots of reciprocal hybrids. Figure S2: Distribution of H3K36me3 levels within and around differentially expressed genes. Figure S3: Experimental validation of methylated DNA regions by genomic bisulfite sequencing. Figure S4: A representative genomic region on maize chromosome 1 showing integrated maps of transcription and epigenetic modifications. Figure S5: Tree view of hierarchical clustering of H3K9ac and H3K36me3 levels. Figure S6: Relationships of variations in H3K36me3 and gene expression between organs and between genotypes. Figure S7: Functional categories of genes upregulated in shoots and roots of hybrids. Figure S8: Correlation of allelic expression bias between shoots and roots of Mo17 ´ B73. Figure S9: Coverage of 21 nt, 22 nt, and 24 nt siRNA clusters in and around protein-coding genes. Figure S10: Distribution of 21 nt and 22 nt siRNA clusters on maize chromosome 1. Figure S11: Correlation between 22 nt siRNAs and DNA-methylation levels at the same genomic loci. [file gb-2013-14-6-r57-S2.PDF]

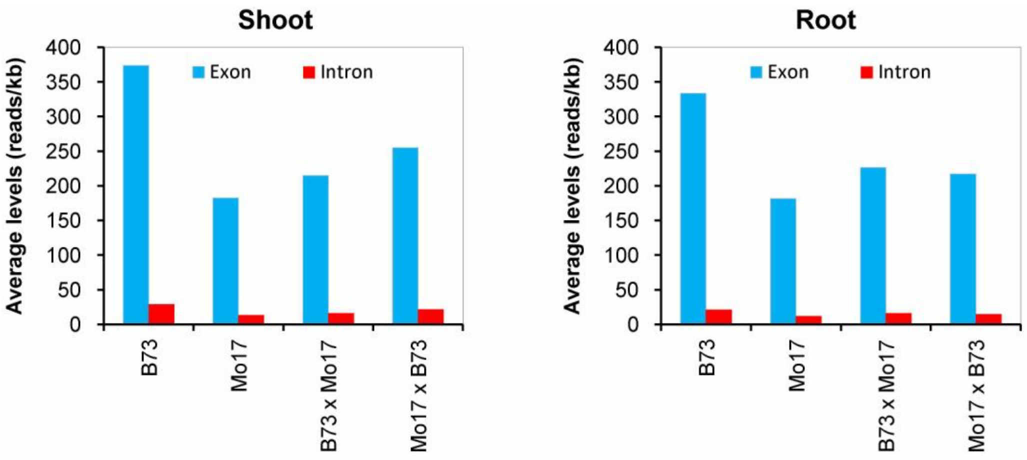

Figure S1. Average levels of exons and introns in shoots and roots of reciprocal hybrids.

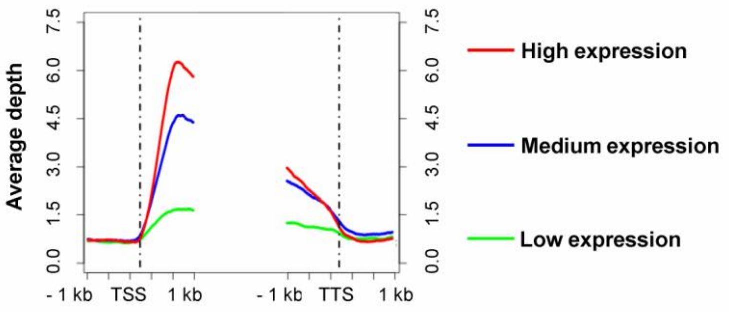

Figure S2. Distribution of H3K36me3 levels within and around differentially expressed genes. The average read coverage of genes with H3K36me3 was plotted (*y*-axis). Genes with detected transcripts were sorted according to their expression levels and further divided into three groups (high, medium and low expression, each with equal number of genes). TSS, Transcription Start Site; TTS, Transcription Termination Site.

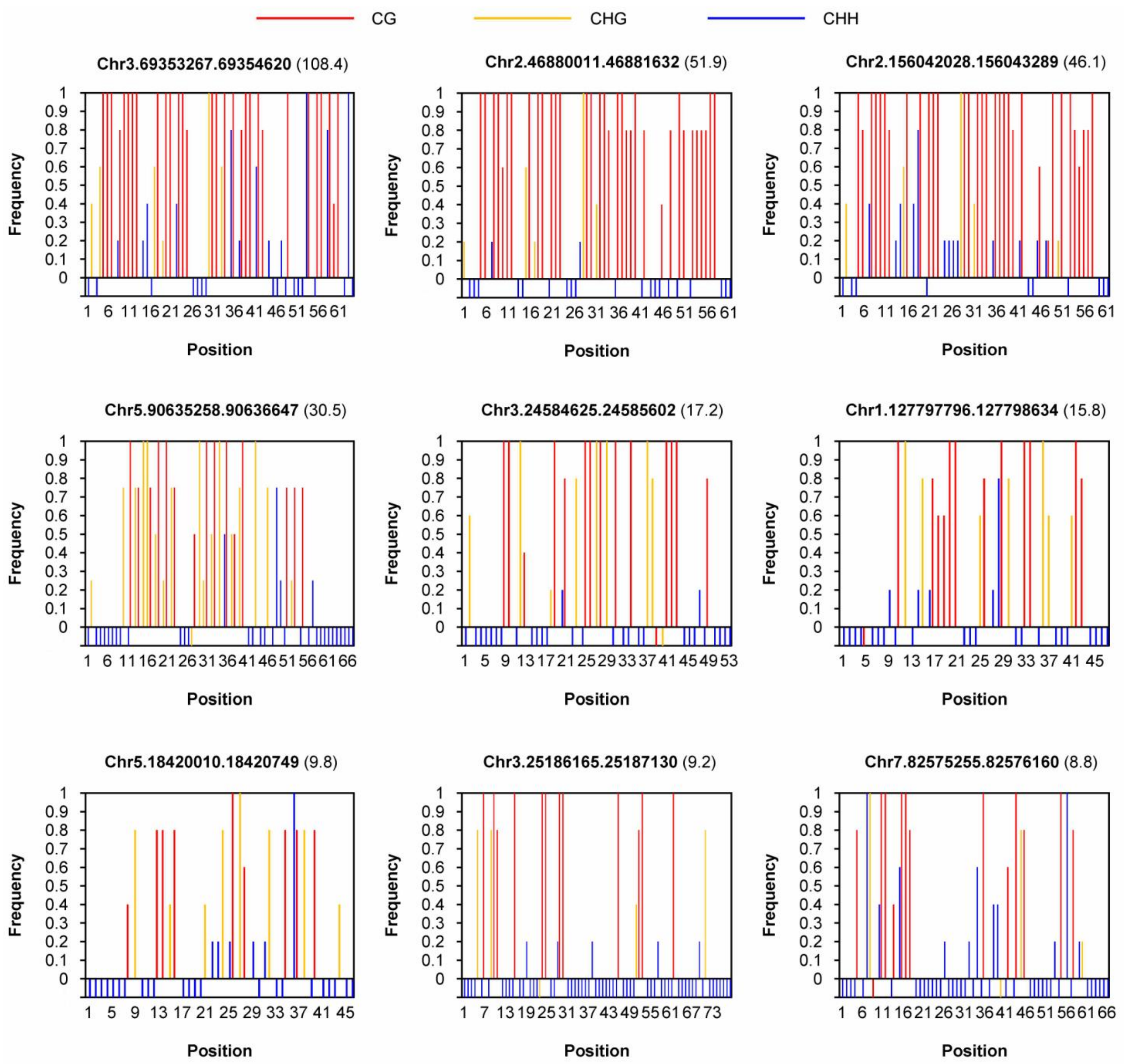

Figure S3. Experimental validation of methylated DNA regions by genomic bisulfite sequencing. Methylated cytosines were detected in the indicated regions from seedling shoots by bisulfite sequencing as previously described [1]. Numbers in brackets are DNA methylation levels detected by *Mcr*BC-seq in this study (in RPKM). CG, CHG, and CHH = methylcytosines detected in the indicated contexts.

1. Li X, Wang X, He K, Ma Y, Su N, He H, Stolc V, Tongprasit W, Jin W, Jiang J, Terzaghi W, Li S, Deng XW: **High-resolution mapping of epigenetic modifications of the rice genome uncovers interplay between DNA methylation, histone methylation, and gene expression.** *Plant Cell* 2008, **20:**259-276.

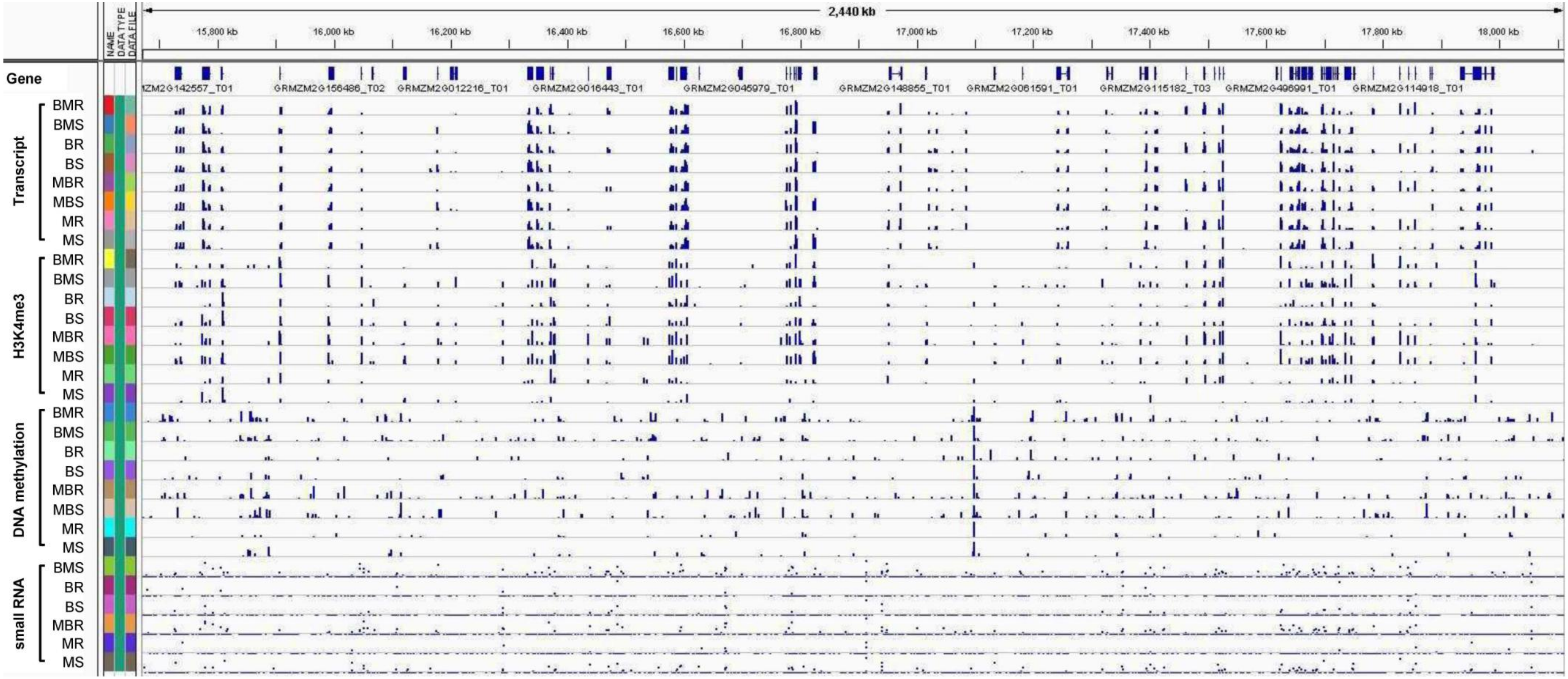

Figure S4. A representative genomic region on maize chromosome 1 showing integrated maps of transcription and epigenetic modifications. B, B73; M, Mo17; BM, B73 x Mo17; MB, Mo17 x B73; S, Shoot; R, Root.

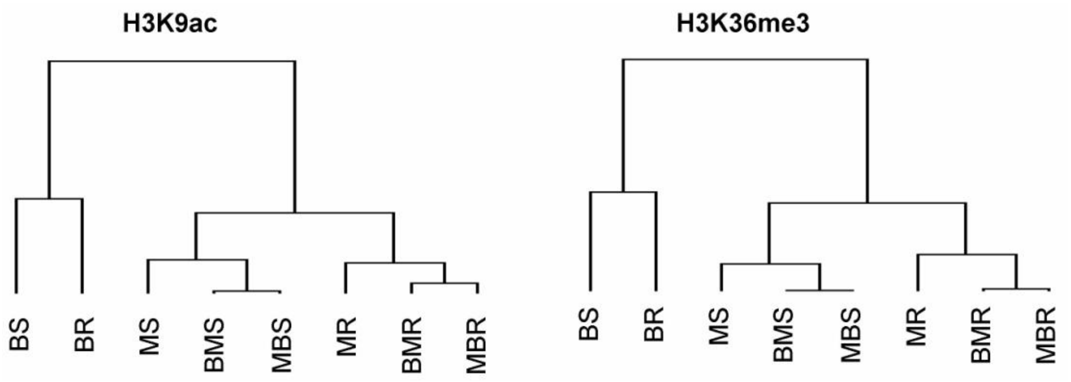

Figure S5. Tree view of hierarchical clustering of H3K9ac and H3K36me3 levels.

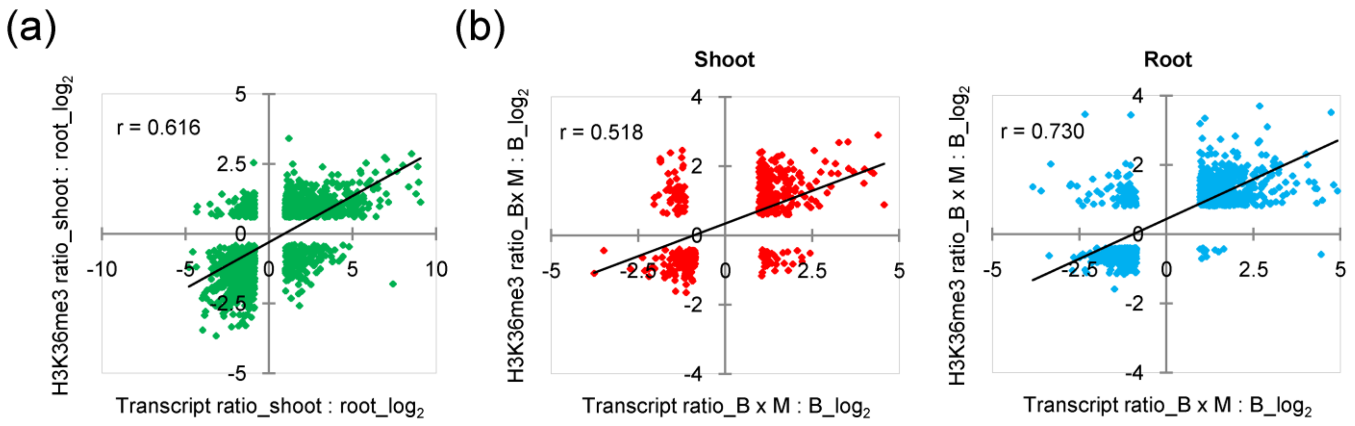

Figure S6. Relationships of variations in H3K36me3 and gene expression between organs and between genotypes. (a) Correlations between differential gene expression (P value < 0.01 and fold change > 2.0) and H3K36me3 (P value < 0.01 and fold change > 1.5) between shoots and roots. **(b)** Correlations between differential gene expression (P value < 0.01 and fold change > 2.0) and H3K36me3 (P value < 0.01 and fold change > 1.5) between hybrids and parents.

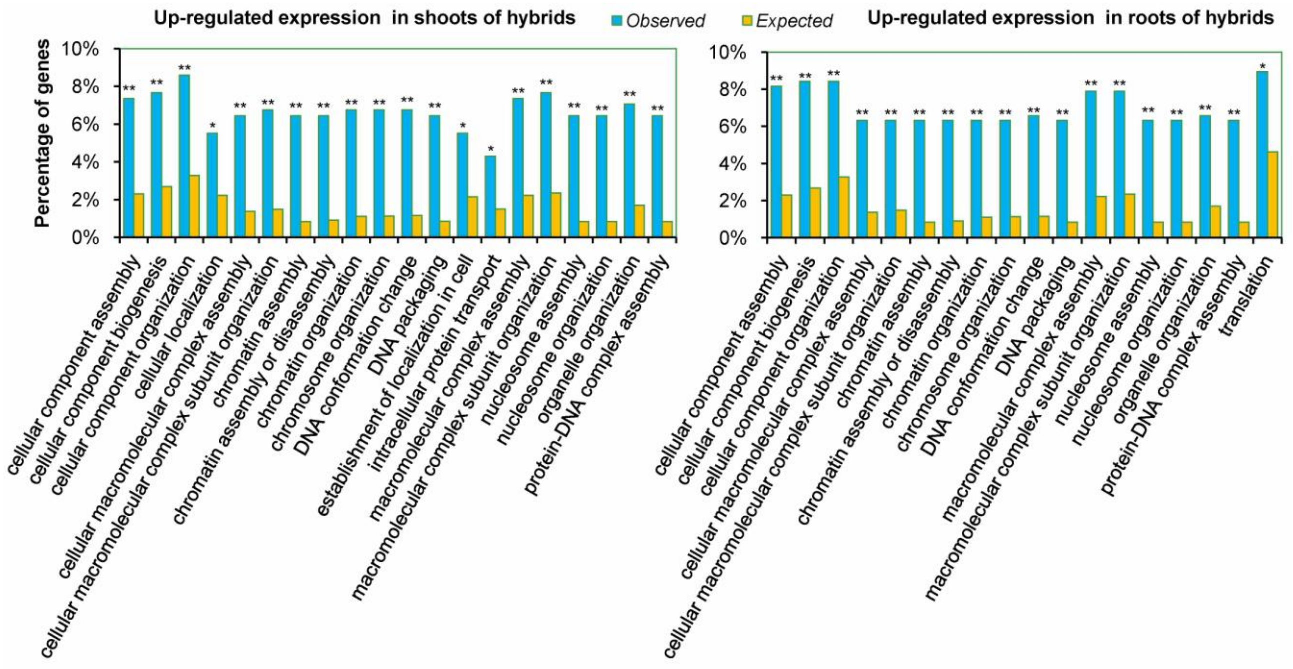

Figure S7. Functional categories of genes up-regulated in shoots and roots of hybrids. * and ** denote significant enrichment of a functional category with FDR adjusted p-value < 0.05 and < 0.01, respectively.

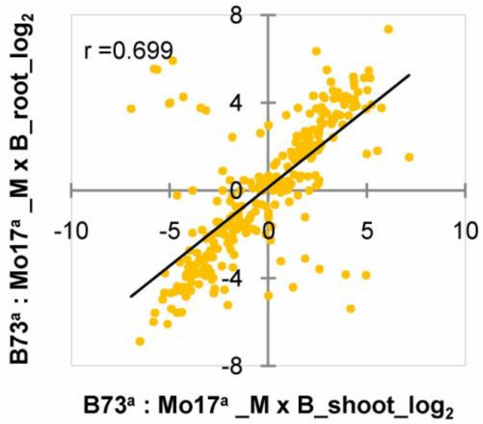

Figure S8. Correlation of allelic expression bias between shoots and roots of Mo17 x B73.

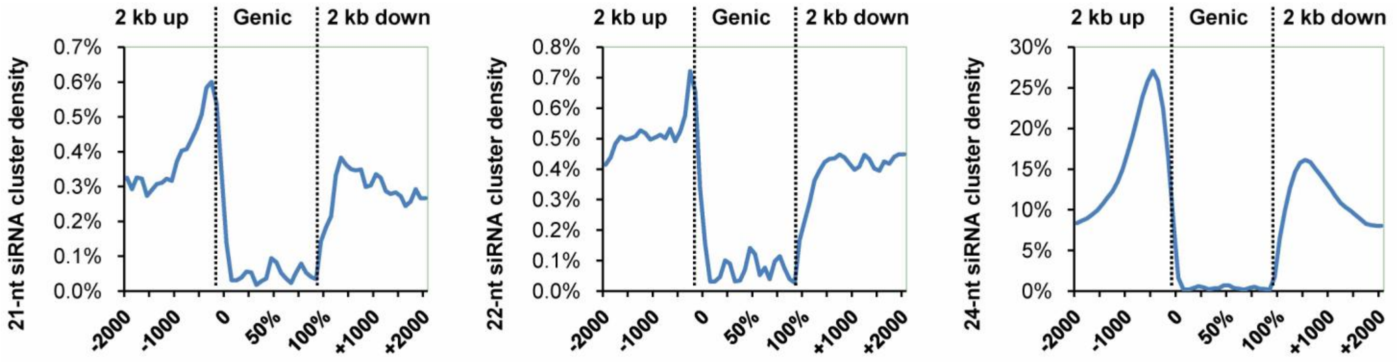

Figure S9. Coverage of 21-, 22- and 24-nt siRNA clusters in and around protein-coding genes.

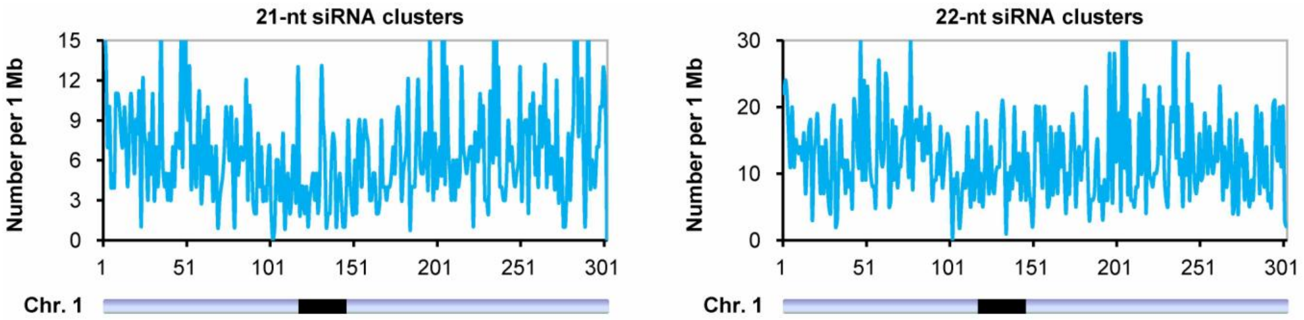

Figure S10. Distribution of 21- and 22-nt siRNA clusters on maize chromosome 1.

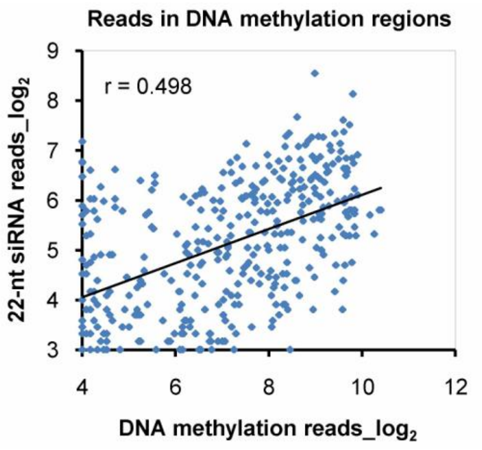

Figure S11. Correlation between 22-nt siRNAs and DNA methylation levels at the same genomic loci.
