# Supplementary material for: Conservation and divergence of transcriptomic and epigenomic variation in maize hybrids
Source: Genome Biol. 2013 Jun 12;14(6):R57. doi: 10.1186/gb-2013-14-6-r57 (PMC3707063; doi:10.1186/gb-2013-14-6-r57)
Supplement: Additional file 4 — Supplemental methods. Details of data processing and analyses. [file gb-2013-14-6-r57-S4.PDF]

Supplemental methods

Data processing and analyses

Raw sequencing reads from all libraries were mapped to the reference genome of the maize inbred line B73 (version 2; ZmB73_RefGen_v2) [http://ftp.maizesequence.org/current/assembly/] [1] using Bowtie software [2]. Sequencing reads from mRNA-seq were also mapped to Mo17 whole-genome shotgun sequences [ftp://ftp.jgi-psf.org/pub/JGI_data/Zea_mays_Mo17/] using the same software. For the data of mRNAs, histone modifications and DNA methylation, up to two mismatches per read were allowed during the alignment procedure. For small RNA data, the 3' adapter sequences were removed by a custom Perl script before alignment, and adapter-trimmed small RNA reads which were perfectly matched to the reference genome were retained. Small RNA sequencing reads matched to tRNAs, rRNAs, small nuclear RNAs (snRNAs) and small nucleolar RNAs (snoRNAs) were also excluded from further analysis. For all sequencing data, the reads mapped equally well to multiple locations in the genome without mismatch or with identical mismatches were assigned to one position at random as previously reported [3]. For each epigenetic modification, genome-matched reads from all eight libraries (from shoots and roots of hybrids and parents) were pooled to identify genomic regions associated with DNA methylation and each histone modification using MACS software [4] with default parameters (bandwidth, 300 bp; mfold, 32; P value < 1.00e-05). The small RNA reads that corresponded to the precursors of all known maize miRNAs from miRBase [http://www.mirbase.org/index.shtml] were used to estimate the expression level of each maize miRNA [5], and the remaining small RNA reads were pooled and used to identify 21-, 22- and 24-nt siRNA clusters, respectively. A siRNA cluster was defined as a genomic region containing a minimum of six small RNA reads, each separated from the nearest neighbor by a maximum of 200 nt.

For all comparisons, read counts were normalized to the aligned reads per kilobase exon model per million mapped reads (RPKM) and the aligned reads per kilobase genic (or genomic) region per million mapped reads (RPKM), respectively, to obtain the relative levels of expression or epigenetic modifications for each gene or modified region. We found that the replicates of our sequencing data are highly correlated (Pearson Correlation Coefficients are from 0.984 to 0.999 for mRNA transcriptomic data and from 0.851 to 0.960 for epigenomic data). We estimated the variance between replicates using DESeq [6] which is based on the negative binomial distribution, and excluded the genes or genomic regions showing significant discrepancy (P value < 0.05) in expression or epigenetic modifications among biological replicates, and then

combined the data from the replicates of each sample to get higher sequencing depth and to detect subtle changes. Differentially expressed genes, modified regions or expressed siRNAs between tissues or among hybrids and parents were identified using DEGseq software [7] which was implemented in R [http://www.r-project.org/] with a selection of random sampling model of reads distribution (MARS, MA-plot-based method with Random Sampling model). Genes, genomic regions or siRNA clusters with detected expression or modifications in both compared samples were included in a pairwise comparison.

The complete genome of maize inbred line B73 was split into 200 bp windows and aligned against the genomic sequence of maize inbred line Mo17 using blastn program with a cutoff of e^{-10} to identify orthologous genomic regions between two parental inbred lines. The annotated maize genes in the B73 genome (release 5b.60 filtered gene set) located in orthologous genomic regions and showing sequence identity of more than 90% with Mo17 were identified as the homologous genes between B73 and Mo17. In each tissue, SNPs were identified by comparing sequencing reads of all homologous genes between B73 and Mo17 with the following criteria: all reads are uniquely matched to both parental genomes; there are at least five sequencing reads covering a SNP position in each parent; all reads from one parent contain the same nucleotide at the SNP position and are different from that at the same position of another parent. Allelic bias in hybrids was identified by determining whether there are significant deviations from binomial distribution (i.e., the allele ratio in hybrids deviated from 1.0) of parental alleles defined by SNPs as described previously [8].

Gene ontology classification provided in Maize Genetics and Genomics Database [http://www.maizegdb.org/] [9] was used to assign a gene to a hierarchical biological process using agriGO, a web-based tool and database for the gene ontology analysis [http://bioinfo.cau.edu.cn/agriGO/] [10], with FDR adjusted P value (Q value) cutoff of 0.05 and 0.01 as the significance threshold.

1. Schnable PS, Ware D, Fulton RS, Stein JC, Wei F, Pasternak S, Liang C, Zhang J, Fulton L, Graves TA, Minx P, Reily AD, Courtney L, Kruchowski SS, Tomlinson C, Strong C, Delehaunty K, Fronick C, Courtney B, Rock SM, Belter E, Du F, Kim K, Abbott RM, Cotton M, Levy A, Marchetto P, Ochoa K, Jackson SM, Gillam B, *et al*: **The B73 maize genome: complexity, diversity, and dynamics.** *Science* 2009, **326:**1112-1115.
2. Langmead B, Trapnell C, Pop M, Salzberg SL: **Ultrafast and memory-efficient alignment of short**

DNA sequences to the human genome. *Genome Biol* 2009, **10:**R25.

3. Wang X, Elling AA, Li X, Li N, Peng Z, He G, Sun H, Qi Y, Liu XS, Deng XW: **Genome-wide and organ-specific landscapes of epigenetic modifications and their relationships to mRNA and small RNA transcriptomes in maize.** *Plant Cell* 2009, **21:**1053-1069.
4. Zhang Y, Liu T, Meyer CA, Eeckhoute J, Johnson DS, Bernstein BE, Nusbaum C, Myers RM, Brown M, Li W, Liu XS: **Model-based analysis of ChIP-Seq (MACS).** *Genome Biol* 2008, **9:**R137.
5. Nobuta K, McCormick K, Nakano M, Meyers BC: **Bioinformatics analysis of small RNAs in plants using next generation sequencing technologies.** *Methods Mol. Biol.* 2009, **592:**89-106.
6. Anders S, Huber W: **Differential expression analysis for sequence count data.** *Genome Biol.* 2010, **11:**R106.
7. Wang L, Feng Z, Wang X, Zhang X: **DEGseq: an R package for identifying differentially expressed genes from RNA-seq data.** *Bioinformatics* 2010, **26:**136-138.
8. He G, Zhu X, Elling AA, Chen L, Wang X, Guo L, Liang M, He H, Zhang H, Chen F, Qi Y, Chen R, Deng XW: **Global epigenetic and transcriptional trends among two rice subspecies and their reciprocal hybrids.** *Plant Cell* 2010, **22:**17-33.
9. Schaeffer ML, Harper LC, Gardiner JM, Andorf CM, Campbell DA, Cannon EK, Sen TZ, Lawrence CJ: **MaizeGDB: curation and outreach go hand-in-hand.** *Database (Oxford)* 2011: bar022.
10. Du Z, Zhou X, Ling Y, Zhang Z, Su Z: **agriGO: a GO analysis toolkit for the agricultural community.** *Nucleic Acids Res* 2010, **38:**W64-70.
